# Rapid Profiling of the Antigen Regions Recognized by Serum Antibodies Using Massively Parallel Sequencing of Antigen-Specific Libraries

**DOI:** 10.1371/journal.pone.0114159

**Published:** 2014-12-04

**Authors:** Maria Domina, Veronica Lanza Cariccio, Salvatore Benfatto, Deborah D'Aliberti, Mario Venza, Erica Borgogni, Flora Castellino, Carmelo Biondo, Daniel D'Andrea, Luigi Grassi, Anna Tramontano, Giuseppe Teti, Franco Felici, Concetta Beninati

**Affiliations:** 1 SPGMB Department, University of Messina, Messina, Italy; 2 SSMCSO Department, University of Messina, Messina, Italy; 3 Novartis Vaccines and Diagnostics, Siena, Italy; 4 Department of Physics, Sapienza University, Rome, Italy; 5 Charybdis Vaccines Srl, Messina, Italy; 6 Department of Biosciences and Territory, University of Molise, Pesche, Isernia, Italy; Imperial College London, United Kingdom

## Abstract

There is a need for techniques capable of identifying the antigenic epitopes targeted by polyclonal antibody responses during deliberate or natural immunization. Although successful, traditional phage library screening is laborious and can map only some of the epitopes. To accelerate and improve epitope identification, we have employed massive sequencing of phage-displayed antigen-specific libraries using the Illumina MiSeq platform. This enabled us to precisely identify the regions of a model antigen, the meningococcal NadA virulence factor, targeted by serum antibodies in vaccinated individuals and to rank hundreds of antigenic fragments according to their immunoreactivity. We found that next generation sequencing can significantly empower the analysis of antigen-specific libraries by allowing simultaneous processing of dozens of library/serum combinations in less than two days, including the time required for antibody-mediated library selection. Moreover, compared with traditional plaque picking, the new technology (named Phage-based Representation OF Immuno-Ligand Epitope Repertoire or PROFILER) provides superior resolution in epitope identification. PROFILER seems ideally suited to streamline and guide rational antigen design, adjuvant selection, and quality control of newly produced vaccines. Furthermore, this method is also susceptible to find important applications in other fields covered by traditional quantitative serology.

## Introduction

Measuring the total concentration of antigen-specific serum antibodies is a fundamental step in the diagnosis of infectious and autoimmune diseases and is used to monitor the efficacy of vaccination, which is the most powerful tool to preserve human health and to reduce the costs of medical care. However, a purely quantitative analysis of serum antibodies is a poor indicator of the complexity of the antibody response, which involves the activation of thousands of different B cell clones and the secretion of a wide variety of antibodies, each directed against a different region of the immunizing antigen(s) [1]. For reasons that are only partially understood, the antibodies induced by any immunizing antigen are not equally directed against the various portions of the antigen molecule [2]. Often, within an antigen, there are regions that are strongly reactive with antibodies (i.e. immunodominant regions) flanked by domains that seem to be partially or completely ignored by the immune system. Anti-microbial vaccination induces the production of a great variety of antigen-specific antibodies, only a minority of which possesses the ability to protect against target infections [2], [3]. In other words, only certain antibodies - those directed against specific “hot spots” of the antigen molecule - have immunoprotective activities. Therefore, it has been proposed that pathogens sometimes adopt the strategy of incorporating, in the context of their virulence factors, immunodominant regions that function as “decoys” by preventing the immune system from targeting the “hot spots” [3]. Although the exact nature and function of such “decoy” epitopes are still ill-defined, it is well established that selective removal of immunodominant, non-protective regions can boost the ability of the antigen to protect against infection after immunization [4]. In view of these considerations it would be helpful, particularly in the course of preclinical studies and clinical trials involving vaccines, to establish whether the immune response is optimally targeted against the antigenic residues crucial for immune-mediated protection. To this end, a method capable of providing a detailed analysis of the fine specificity of vaccine-induced antibody repertoires would be useful to guide rational antigen design and selection of appropriate adjuvants. Indeed, the ability of certain adjuvants to broaden the antibody repertoire and to provide extended coverage of antigen regions targeted by polyclonal responses is becoming increasingly clear [5], [6]. Moreover, because the spectrum of antibody specificities varies with age and physiology, repertoire profiles may be useful to specifically tailor vaccine formulations for different age groups and for high-risk populations [7], [8], [9].

The recent development of high-throughput methods for repertoire data collection - from single cell mass spectroscopy and multicolor flow cytometry to massively parallel sequencing of immunoglobulin transcripts - offers today an opportunity to analyze large samples of lymphocyte repertoires [10], [11], [12]. Although these methods provide extensive information regarding the diversity of clonotypes and immunoglobulin gene usage, they have limited usefulness, by their nature, in sampling the antibody repertoire in terms of epitope specificity. Libraries consisting of phage particles or cells expressing on their surface peptides of various lengths have been widely used in epitope mapping [13], [14], [15], [16]. These techniques are, however, labor-intensive, time consuming and can identify only a limited number of epitopes. We describe here a novel approach, based on the combined use of phage-displayed antigen-specific libraries and massive parallel sequencing of the entire population of affinity-selected phages. This approach, that we named PROFILER (for Phage-based Representation OF ImmunoLigand Epitope Repertoire), generates a high-resolution profile of antigen-specific antibody repertoires in less than two days. As a model antigen we used here NadA [17], one of the 4 components of the 4CMenB anti-meningococcal vaccine (Bexsero). We first generated a lambda phage displayed library in which individual phage particles display on their surface different NadA fragments of various lengths, followed by selection with sera from Bexsero-immunized volunteers and analysis through the Illumina MiSeq platform.

## Results

### Library sequencing

**Figure 1:**
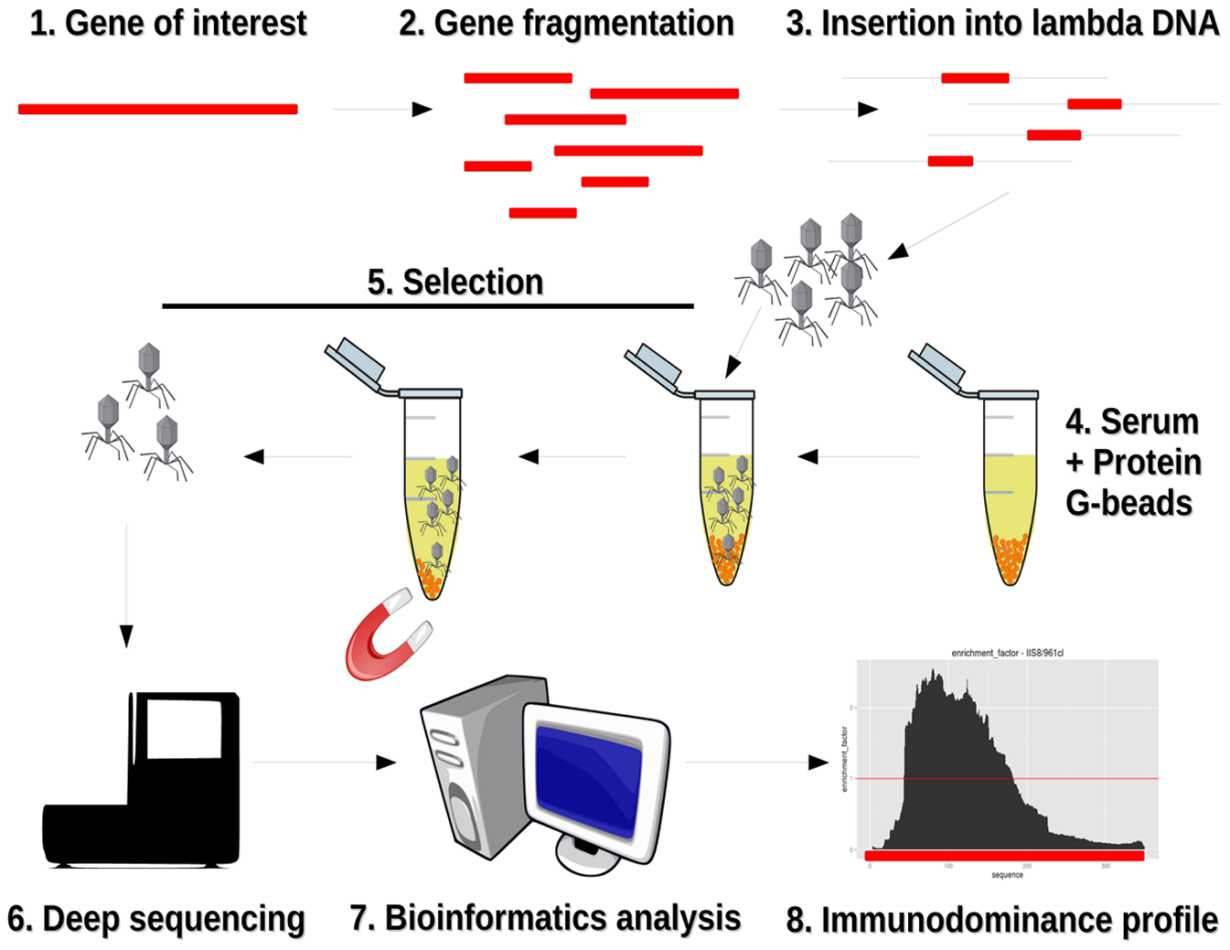
Schematic outline of the epitope mapping approach. The gene encoding the antigen is fragmented by DNAse digestion and the gene fragments are inserted into lambda phage vectors. The phage library is mixed with immune serum and phage particles binding to immunoglobulins are separated using Protein-G coated magnetic beads. The inserts of the phage population obtained after selection are massively sequenced and compared with those of the original unselected library using an *ad hoc* developed software which identifies the region(s) of the antigen targeted by serum antibodies.

The gene-specific library that was used in this study is composed of phage particles carrying, as recombinant inserts, fragments of the *nadA* gene of group B *N. meningiditis* (Fig. 1). Since the sequence of this gene is known, the entire sequence of the inserts can be inferred from the relatively short (150 bp) reads obtained at each end of insert-containing amplicons using Illumina MiSeq paired end sequencing. After quality control of the sequencing output and demultiplexing of the data, FASTQ raw files were generated by the Illumina MiSeq sequencer and processed with Perl scripts generated in house, which list all sequences and divides them into “empty”, “natural frame” and “not natural frame” categories. “Natural frame” sequences are translated into amino acid sequences and analyzed for copy number, length and position along the sequence of the protein antigen.

### Characteristics of the library

By sequencing a few hundred clones by the Sanger method, only some of the properties of a library can be inferred. Using the Illumina MiSeq, instead, we were able to sequence thousands of clones of the *nadA*-specific library and assess, thereby, its quality and diversity in depth. First, we found that 4.8% percent of all sequences containing *nadA* fragments were “natural frame”, i.e. fulfilled the requirements to be expressed on the phage surface as authentic NadA peptides (not shown). This percentage is close to the expected 1/18 (5.6%) value, calculated as the probability that a gene fragment is randomly cloned as an insert in the natural frame at the N-terminus of the lambda phage capsid protein D encoding sequence.

**Figure 2:**
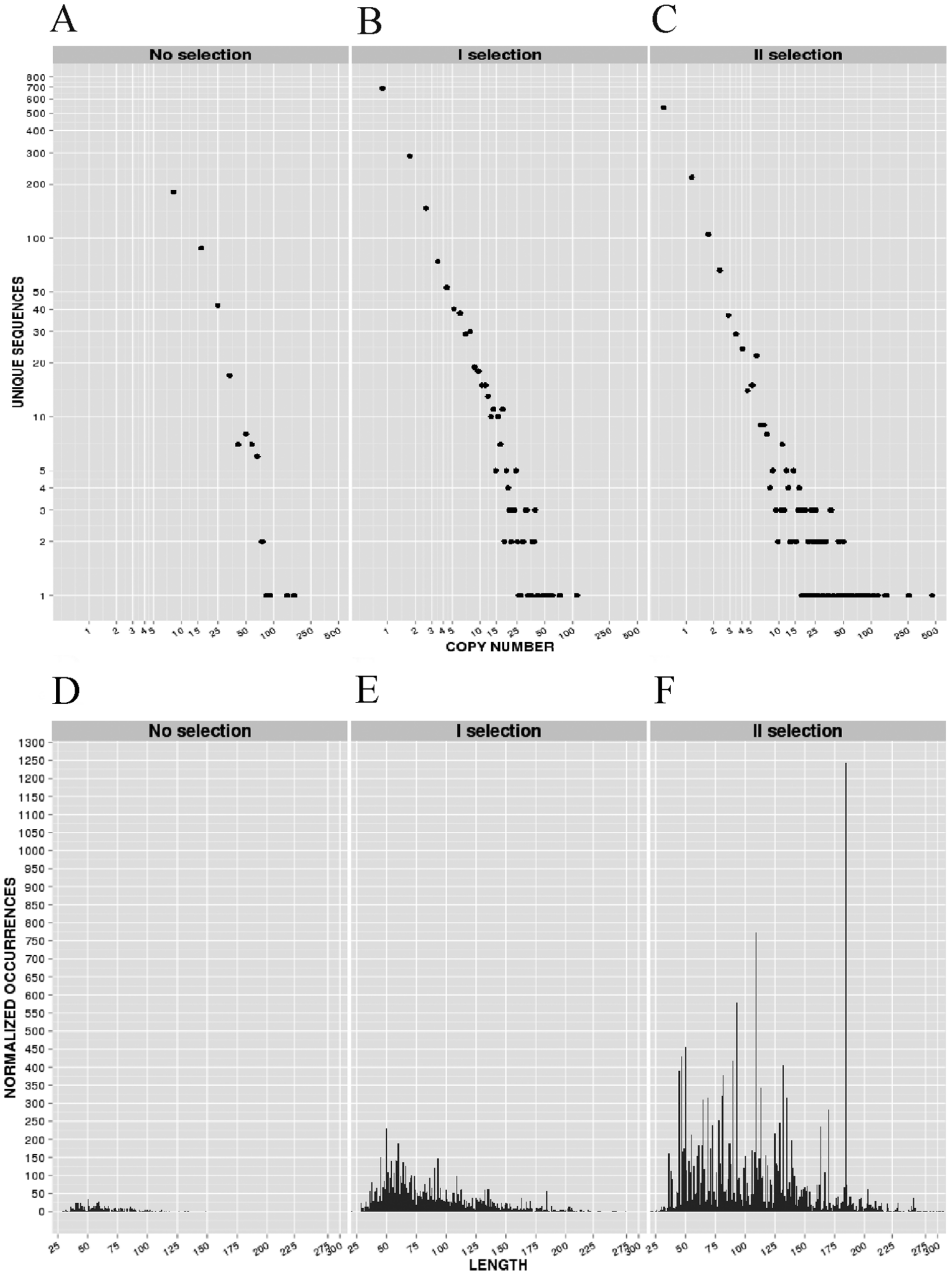
Properties of the antigen-specific phage library before and after selection with a pool of serum samples from volunteers immunized with the Bexero anti-MenB vaccine. A–C, abundance of “natural frame” *nadA* fragments in the library before (A) and after the first and second rounds of selection (B and C, respectively). Each point represents the number of unique fragments (vertical axis) displaying the number of copies indicated in the horizontal axis; D–F, *nadA* fragment length distribution before (D) and after the first and second rounds of selection (E and F, respectively).

**Figure 3:**
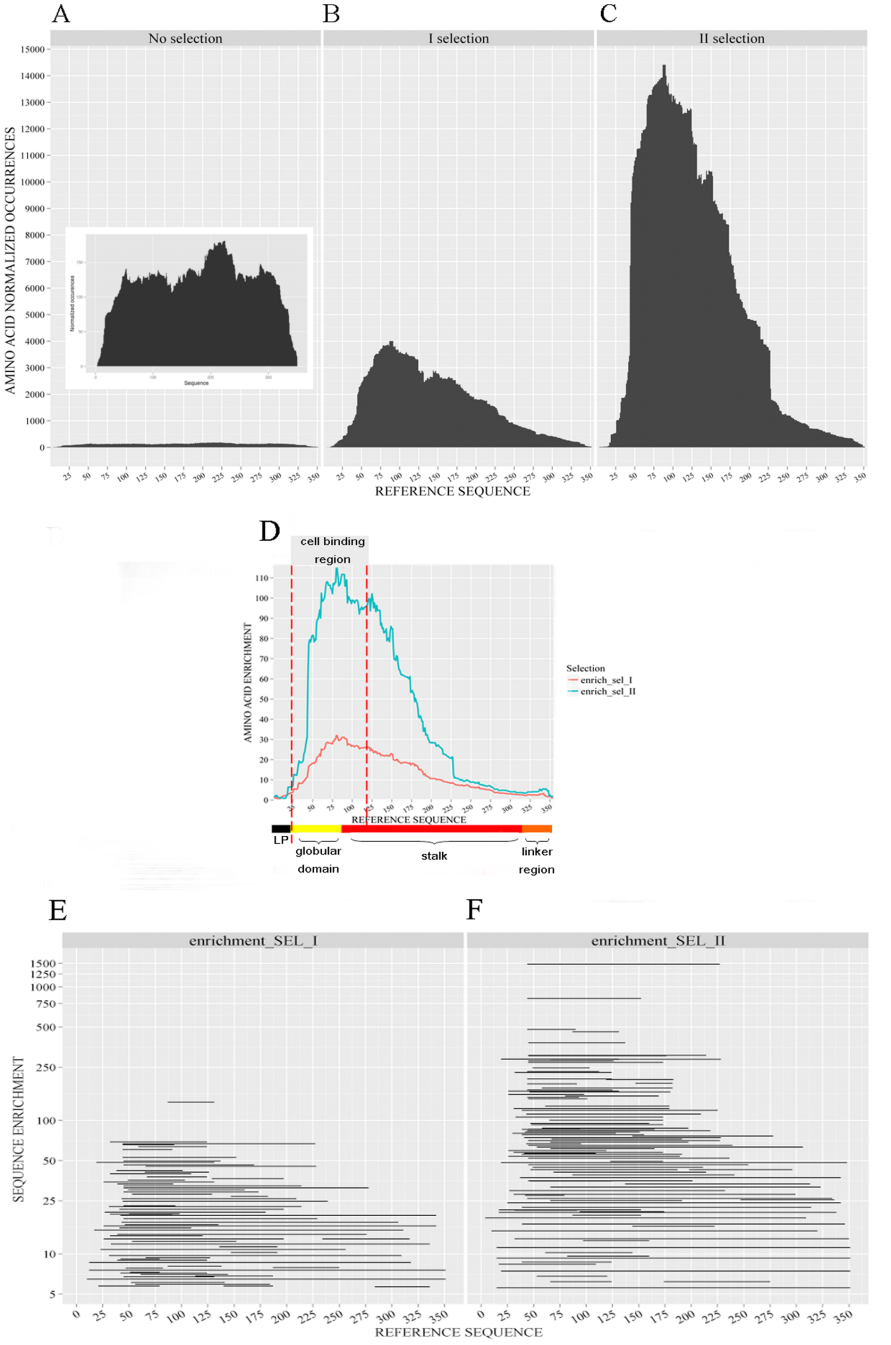
Enrichment of phage clones predicted to display authentic NadA fragments on their surface after selection with a serum pool from volunteers immunized with the Bexero vaccine. Frequency values reported in the vertical axis in panels A–C refer to the occurrence, per single amino acid position, of sequences predicted to express authentic NadA fragments, relative to those predicted to express irrelevant or no polypeptides. The inset in figure A reports the same data with a higher y-axis magnification. The horizontal axis reports the amino acid positions of the translated NadA sequence. A, unselected library; B and C, library outputs after one and two rounds of selection, respectively. D, Cumulative enrichment factors for each amino acid position derived from NadA fragments obtained after one (blue line) and two (red line) rounds of selection; colored bars in the horizontal axis refer to NadA domains; the area between the dashed vertical lines correspond to the cell binding region of NadA [18]. E and F, enrichment factors of NadA fragments after one and two rounds of selection, respectively. Only the fragments laying in the upper quartile of enrichment factors values are shown.

A good antigen-specific library should contain a large diversity of random fragments of the gene of interest, while marked overrepresentation of some unique sequences is an indicator of lower quality. Since no unique fragments were found in copy numbers higher than 20 in the unselected library and 90% of sequences were found in copy numbers of <5 (Fig. 2A), no major overrepresentation of specific antigen fragments was found in the unselected library. The length distribution of expressed authentic NadA fragments had a mean value of 85 amino acid, ranging from 29 to 210 (Fig. 2D). Therefore, NadA fragments were only slightly shorter than expected from the average length (approximately 300bp) of the size-selected DNA fragments cloned into the phage vector. As shown in Fig. 3A, these fragments were evenly distributed along the entire sequence of the protein, with no major over- or under-representations of specific regions. Minor under-representation of short amino acid stretches at either end of the protein was expected based on library construction, which involved fragmentation of the *nadA* gene by DNase I digestion. In conclusion, the analysis of next generation sequencing data enabled an in-depth evaluation of the characteristics of the NadA library and demonstrated that its fundamental properties were in agreement with its design.

### Affinity selection with immune human sera

During an antibody-mediated selection process, phage particles displaying specific antigenic fragments are expected to be progressively enriched, while the remaining phage clones should decrease in number and be eventually lost. To follow the process of antibody-mediated selection, two rounds of phage selection were performed on the *nadA* library using a pool of sera obtained from adults immunized with the 4CMenB Bexsero vaccine, which contains recombinant NadA as one of the antigens. For each selection, over 10^4^ sequences were obtained and analyzed. During the selection, there was a dramatic, progressive increase in the frequency of “natural frame” sequences, as it can be appreciated by the black areas in Fig. 3A–C. This indicated that phage particles displaying authentic NadA fragments had been selectively enriched by the NadA-specific antibodies present in the immune sera, while those carrying “not natural frame” or no inserts rapidly decreased in numbers.

To further characterize the enrichment process, we analyzed “natural frame” sequences for copy numbers and length. As expected in a typical phage display experiment, the frequency of the most enriched polypeptides dramatically increased during subsequent rounds of selection (Fig. 2B and C), indicating a rapid convergence towards specific fragments during the selection process. Moreover, the NadA fragments obtained after selection were on average longer than those of the unselected library (Fig. 2D–F) with mean fragment lengths of, respectively, 85 and 141 amino acids before and after two selection rounds. The reason for this effect is not entirely clear, but it's likely related to the more frequent occurrence in the longer fragments of conformational epitopes, of multiple epitopes or of both.

Among the “natural frame” sequences, those corresponding to a specific region of the NadA molecule seemed to be over-represented in the affinity-selected library (Fig. 3A–C). To more precisely quantitative this feature, we calculated the “enrichment factor” for each “natural frame” sequence as the ratio between the occurrence of that sequence in the affinity-selected phage population and its occurrence in the unselected library, as detailed in Materials and Methods. By displaying the enrichment factor values as a function of the amino acid positions along the NadA sequence (Fig. 3D), it was possible to unambiguously identify NadA regions that were enriched by the antibody-mediated selection process. With the exception of the first 15 amino acids, these regions corresponded to the portion of the NadA molecule involved in adhesion to host cells [Fig. 3F; [18]].

The position along the NadA sequence of the most enriched fragments is shown in Fig. 3E and F. To demonstrate that the enrichment of these fragments was actually linked to their reactivity with the antibodies present the serum pool used for selection, individual clones were isolated from the library and tested in a phage ELISA assay. As shown in Table 1, fragments corresponding to clones with phage ELISA titers of 400 or higher all mapped to the area of the protein, with enrichment factor values above 1, shown in Figure 3F. In contrast, clones with fragments that lied outside of this area had phage ELISA titers of 200 or lower. Collectively, these data indicated that massive sequencing of antigen-specific libraries allows an accurate characterization of the antibody-mediated selection process, including identification of the antigen region(s) bearing immunodominant epitopes.

**Table 1:** Immunoreactivity of selected phage clones corresponding to different fragments of the *N. meningitidis* NadA antigen.

Clone ID	Start-stop amino acid	Enrichment factor	Titer
15_B12	45–137	382	1600
26_H07	45–214	307	1600
97_A07	44–227	1481	1600
100_E02	44–151	5	800
26_A01	44–93	149	800
21_D09	152–193	−5	400
Cl_134	60–123	−4	400
16_B09	125–191	−4	400
CL_178	06–94	−3	200
961_1_F1	285–339	−4	200
11_H07	201–259	−4	200
14_D08	279–320	−4	100
21_B07	10–59	−4	100
12_F04	190–225	−5	100

### Comparison with the traditional method

Next, we compared the novel massive sequencing approach with traditional single clone sequencing. To this end, we randomly picked 50 plaques from the output of the second round of selection and sequenced them by the traditional Sanger method after PCR amplification. Several labor-intensive days were required to analyze a single selection output, including the time needed to interpret the sequences and analyze the results. In contrast, using PROFILER sequencing, two days were sufficient to complete sequencing and data analysis. Moreover, the new technology allows coverage of dozens, as opposed to one or few, output phage populations (originating for example, from different library/serum sample combinations) in a single multiplexed MiSeq run.

**Figure 4:**
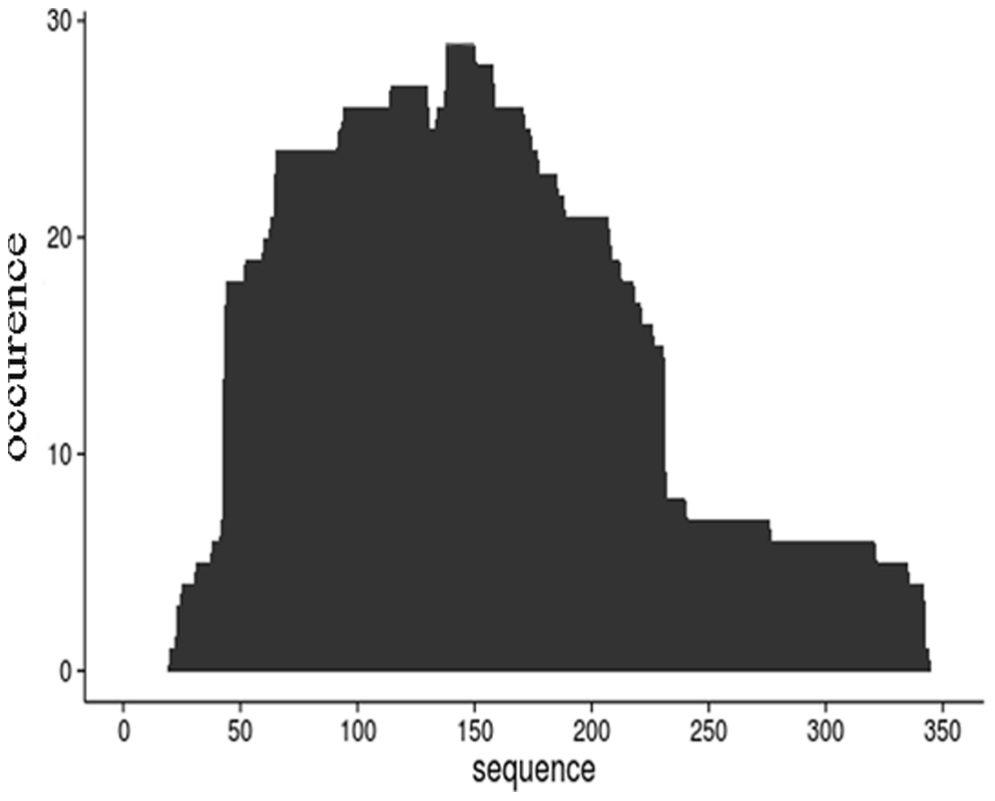
Occurrence of cumulative amino acid positions in NadA fragments obtained after two rounds of selection, as determined by traditional random picking of 50 plaques followed by Sanger sequencing of individual clones.

When data from Sanger sequencing were plotted in the form of cumulative occurrence of single amino acid positions in the NadA molecule, the overall shape of the graph was roughly similar to that of the previously obtained PROFILER graph (confront Fig. 3D and Fig. 4). However, analysis of the fragments identified by the traditional method revealed that 41 of the 50 sequences were found only once, leaving some uncertainty about their actual degree of enrichment after the selection process. Moreover, only 11 of the 50 sequences obtained by the traditional technology were derived from the 10 most abundant phage populations, as identified by PROFILER (not shown). Since these 11 sequences represented four clones only, six of the ten most abundant sequences were missed by the conventional approach. Because the ability to identify short, in addition to long, epitope-containing fragments is useful to increase the resolution of epitope mapping methods, we also compared the traditional and the new method for their frequencies of short sequences in the identified fragments. Notably, “short” (i.e. <50 amino acid long) fragments accounted for roughly 25% of the sequences identified by PROFILER (Fig. 3F), while such fragments were not found among the 50 Sanger clones (not shown). Overall, these data indicated that the traditional technique suffers from limitations stemming from its inability to relate the occurrence of the fragments obtained after affinity selection with that observed in the unselected library, and to estimate, therefore, the actual enrichment of these sequences. For example, only a few unequivocally enriched sequences were identified by the traditional method, while dozens of such sequences were identified using the PROFILER technology. For the same reasons, identification of the antigen regions that are depleted by affinity selection, which may be of particular interest in vaccine development, as it will be discussed below, could not be accomplished by the traditional technique.

## Discussion

We show here that massively parallel sequencing can significantly empower the analysis of phage-displayed antigen-specific libraries during selection with serum antibodies. From a technical standpoint, our data confirm previous studies showing that next generation sequencing can improve the resolution of methods using phage or ribosome display libraries [19], [20], [21], [22], [23], [24]. However, while most of these studies focused on the discovery of peptide [19], [20], [21], [24] and antibody [22] ligands for various targets, the present report deals with the identification of the antigen regions preferentially targeted by polyclonal responses. Moreover, our library, as well as the one used by Larman *et al*. [23], contained sequences obtained from natural repertories, while most of the studies cited above used random peptides libraries, whose results are sometimes difficult to interpret particularly when used in conjunction with polyclonal sera. We believe that, particularly for our application and the one described by Larman *et al*. [23], the use of next generation sequencing may be instrumental not only in increasing the resolution of the assay, but also in moving it from research to clinical laboratories. In this respect, the PROFILER technology, seems amenable to applications in all fields of serology, including the diagnosis of infectious, autoimmune and neoplastic diseases. Serology has been traditionally concerned with determining the *amount* of antibodies directed against a chosen antigen. We show here that - by combining the versatility of phage display with the power of next generation sequencing - it is also possible to obtain, in a time frame close to that of traditional serological techniques (i.e. within two days), precise *qualitative* and *quantitative* information about the regions of the antigen molecule that are targeted by a serum antibody response. Clearly, future studies will be needed to ascertain whether PROFILER will be useful in diagnostic testing and whether specific immunodominance patterns can be linked to well-defined pathologic features or to vaccine efficacy.

There is a great need, in vaccine research and diagnostic medicine, for rapid and simple methods to identify B cell epitopes [25]. Moreover, epitope characterization is important in the context of drug design [26]. The most frequently used epitope mapping method involves scanning with overlapping, chemically synthesized peptides to identify the linear epitopes of an antigen [27]. A limitation of this technique is its relative inability to detect conformational and/or discontinuous epitopes, which represent more than 90% of B cell epitopes [28], [29]. More informative approaches for epitope characterization involve mutagenesis of antigen residues (e.g. by alanine scanning) or determination of the three-dimensional structure of the bound complex, but such procedures are costly, not always successful and not practically applicable to serum samples, but only to monoclonal reagents. Libraries of peptides displayed on the surface of phages or unicellular organisms are an efficient alternative for epitope mapping. Such libraries, which might comprise overlapping fragments of the antigen or totally random peptides, are subjected to repeated rounds of antibody-mediated affinity selection followed by immunoscreening and/or sequencing of selected clones. The entire process is laborious, requires at least few weeks to be completed and, when conventional approaches are used, can only lead to the characterization of a few dozens phage clones.

The PROFILER technology described here is a comprehensive method for the identification of immunoreactive epitope-bearing protein fragments and for the analysis of the epitope repertoire of antibody responses. This technique merges the efficiency of antigen-specific phage display libraries with the power of next generation sequencing, providing a highly streamlined approach for epitope mapping of both monoclonal and polyclonal antibodies. In combination with accurate computational data analysis for data interpretation, PROFILER can provide a detailed map of epitope-bearing antigen fragments recognized by dozens of individual serum samples in a two-day time frame. Both short amino acid stretches and extended (up to 220 amino acid long) epitope-bearing fragments can be identified. This exemplifies the potential of the technology to map both conformational/discontinuous epitopes, which can be retained only in long fragments, and linear ones, which can be precisely mapped using shorter inserts. In addition, the ability to read several hundred thousands, as opposed to one or two hundred, sequences results in unprecedented resolution in epitope identification compared to the traditional method. Direct comparison with the latter indicated that the majority of the clones enriched by affinity selection rounds would have been lost by traditional Sanger analysis. Notably, PROFILER was also able to discriminate between weak epitopes and more dominant ones and to rank antigenic fragments for their immunoreactivity.

The ability of PROFILER to sequence in its entirety the unselected library also allowed the identification of the antigen fragments that were rapidly lost during the affinity selection process. These apparently non-immunodominant protein regions may be of particular interest as vaccine components, as indicated by recent evidence obtained with streptococcal antigens [4], [30], [31]. The potential importance of such non-immunodominant regions is also exemplified by the discovery of protective HIV epitopes that are poorly immunogenic in the context of the native envelope glycoproteins [32]. Based on this information, targeted amino acid substitutions were successful in “opening” and stabilizing a protective, non-immunodominant epitope [33], [34], [35], [36]. Moreover, it was possible to block antibody binding to undesired epitopes functioning as decoys that diverted antibodies responses away from neutralizing epitopes [37], [38].

It is easy to see, from the case study presented here, how PROFILER could be used in the future to guide and streamline rational antigen design. For instance, PROFILER-based analysis of the epitopes recognized (or ignored) by immune sera, in comparison with those bound by protective and non-protective monoclonal antibodies, could be used to rapidly establish whether the polyclonal response (either in individuals or in selected populations) is missing potentially protective targets. In subsequent steps, PROFILER could be used to quickly screen the responses generated by a variety of mutated antigens and to select, thereby, the most promising candidates. In addition, a variety of adjuvants could be rapidly tested to select the ones capable of re-directing the polyclonal response towards the desired epitopes or of broadening the response for increased coverage against multiple variants of the antigen.

In conclusion, massive sequencing of antigen-specific phage-display libraries before and after antibody selection was developed into a highly streamlined method to precisely map the B cell epitopes recognized during an immune response. This new technique, named PROFILER, therefore appears ideally suited to monitor the antibody repertoire of individuals in response to disease-associated antigens and to guide the rational design of drugs or vaccine candidates through epitope mapping.

## Materials and Methods

### Sera

Twenty-one serum samples from healthy volunteers vaccinated with the 4CMenB vaccine (Bexsero) in the course of a phase I clinical trial [39] were provided by Novartis Vaccines and Diagnostics (NDV, Siena, Italy). Samples were pooled together and used for the selection procedures using a library specific for NadA, one of the 3 recombinant proteins that are included in the vaccine (see below).

### NadA library construction and affinity selection

The steps involved in library construction, selection and analysis are schematically outlined in Fig. 1. A lambda-displayed library containing fragments of the *Neisseria meningitidis* group B *nad*A gene was used. The gene was amplified from an expression plasmid used to produce the recombinant antigen (peT 21b, kindly provided by NVD). This plasmid contains a fragment encompassing the whole length of the *nadA* gene with the exception of the portion encoding for the C terminal hydrophobic membrane anchor of NadA (17). To amplify *nadA* from the expression plasmid we used the primers 961cL forward (5′-AAACACTTTCCATCCAAAGTACTGACCAC-3′) and 961cL reverse (5′-ACCCACGTTGTAAGGTTGGAACAGAC-3′) that amplified a 1047 nucleotide-long fragment, which was then purified and DNase digested as previously described [30], [40]. After excising a band with the approximate size of 300 bp from an agarose gel, 30 ng of DNA were cloned in a lambda vector [λKM4, [41]]. The display library was then packaged *in vitro* using the Gigapack III Gold Packaging Extract (Stratagene), generating approximately 6×10^4^ independent phage clones. Recombinant inserts from single clones were analyzed by PCR amplification (primers K47 5′-GGGCACTCGACCGGAATTATCG-3′ and K48 5′-GTATGAGCCGGGTCACTGTTG-3′), as described [41].

Affinity selection of the library with human sera was performed as described previously [41]. Briefly, magnetic beads linked to Protein G (Dynabeads Protein-G; Dynal) were incubated with library-serum mixtures for 1 h at RT under agitation and washed 10 times with 1 ml of washing solution (1X PBS, 1% Triton, 10 mM MgSO_4_). The bound phage particles were recovered by infection of LE392 cells added directly to the beads. After a 20-minute incubation, 10 ml of molten NZY-top agar (48°C) was added to the mixture of infected cells and immediately poured onto NZY plates (15 cm). After overnight incubation, the phage particles were harvested by gentle agitation with 15 ml of SM buffer for 4 hours at 4°C. The phage particles were purified by PEG/NaCl precipitation and stored at 1/10 of the initial volume or used for subsequent selection rounds.

### Sample preparation for Illumina MiSeq sequencing

In order to amplify the phage inserts and to add the adaptors required for sequencing on the Illumina MiSeq platform (www.illumina.com/systems/miseq.ilmn), library pools obtained before or after selection were first amplified in *E. coli*, and the lysates were subjected to polyethylene-glycol NaCl precipitation, as described [41], [42]. Purified suspensions corresponding to 1×10^6^ phage particles were added to a PCR mix containing the following primers:

≠293: TCGTCGGCAGCGTCAGATGTGTATAAGAGACAGCGATTAAATAAGG and ≠294: GTCTCGTGGGCTCGGAGATGTGTATAAGAGACAGGTAATGGGTAAAG.

The above primers contain subsequences specific for invariant regions of the λKM4 vector that are located, respectively, upstream and downstream of the inserts. These primers also include, at their 5′ ends, Illumina adaptor subsequences required for a second amplification step (see below). The first amplification involved initial denaturation (94°C for 5 min) followed by five cycles of denaturation (94°C for 30 sec), annealing (30°C for 30 sec and 50°C for 2 min) and extension (72°C for 1 min) and fifteen cycles of denaturation (94°C for sec) annealing (60°C for 30 sec) and extension (72°C for 1 min).

The PCR products were then purified using the QIAquick kit (Qiagen, cat.28106). The eluted DNA was quantified using the Quant-iT DNA Assay Kit (Life Technologies, Q33130) and a microplate fluorescence reader (Tecan Infinite 200 PRO). Samples were stored at −20°C before preparation for sequencing, which involved a second PCR step (12 cycles) to add the adaptors necessary for sequencing and the barcodes (i.e. the indexes for multiplexing). Briefly, 5 ng of purified DNA were added to different primers containing adapters (P5 or P7) necessary for binding to the Illumina MiSeq flow cell and different combinations of 8 nucleotides to be used as indexes using the Nextera PCR master Mix kit according to the manufacturer's instructions. Purity, concentration and length of PCR products were checked using a LabChip XT system (Caliper, LifeSciences, PerkinElmer). Next, equal volumes of normalized library were combined and heat-denatured prior to MiSeq sequencing according to manufacturer's instructions. Since our sequencing fragments were identical in the first 48/49 nucleotides and low library diversity does not allow proper matrix calculation by Illumina sequencers, it was necessary to also include (or “spike-in”) a high-diversity library (PhiX library, FC-110-3001, Illumina) accounting for approximately 10% of total sequences. Sequencing was performed using the Miseq Nano kitv2 and paired end, 150 bp-long reads (Illumina, MS-103-1001).

### Data analysis

Sequence data from the insert amplicons were processed with an *ad hoc* pipeline made of Perl scripts. It gives as a result a list of all univocally definable (or “unique”) sequences, classified into one of the following three categories: a) “empty” i.e. sequences lacking fragments of the antigen-encoding gene (*nadA* sequences in this study); b) “natural frame”, i.e. sequences bearing fragments of the antigen-encoding gene, the products of which are predicted (because in the correct orientation and reading frame with respect to the recombinant insert sequence) to display authentic peptide fragments of the antigen on the phage surface; c) “not natural frame”, i.e. sequences bearing fragments of the antigen-encoding gene that are not expressed on the phage surface, or that express peptides corresponding to a non-authentic reading frame because of frame shifts.

The paired-end reads are expected to contain in the following order: vector, adapter and insert. The pipeline checks whether these elements are present in each read in the correct order (forward in the left reads and reverse in the right ones). Subsequently for each pair of reads, short regions, named anchors, are extracted from the insert in proximity of the adapter. The complete insert is then defined as the region of the reference gene included between the two anchors. The nucleotide sequence of the insert is translated into amino acids starting from the first ATG codon. Peptides that do not continue in frame with the phage protein sequence cannot be expressed on the phage surface. We label these as “not in downstream frame” and filter them out. The remaining sequences that can be aligned to the reference amino acid sequence of the target are classified as “natural frame”. For each of these fragments the scripts report the following attributes: a) copy number; b) amino acid sequence and length; c) start and end position of the corresponding amino acid sequence in the NadA protein.

In order to follow the enrichment of “natural frame” inserts during the selection process (Fig. 2 A–C) we normalized the counts of each “natural frame” insert by dividing them by the total number of sequenced reads in a given experiment and multiplying them for the mean value of sequenced reads in all the experiments [43]. We calculated the occurrence of each amino acid of the NadA sequence by summing the counts of all inserts in the corresponding position. The “enrichment factor” for each amino acid residue of the “natural frame” sequences was calculated as the ratio between the occurrence of the residue in the affinity-selected phage population and its occurrence in the unselected library, after adding a pseudocount of 1 [44], [45] to the counts for each position.

### Phage ELISA

Selected phage clones, obtained by plaque picking after affinity selection, were tested in phage-ELISA assay using the serum pool employed for selection, as described elsewhere [41]. Briefly, multiwell plates (Immunoplate Maxisorp, Nunc) were coated overnight at 4°C with 100 µl/well of polyclonal anti-lambda antibodies (1 µg/ml) in coating buffer (50 mM NaHCO_3_, pH 9.6). Plates were then incubated at 37°C for 1 h with 200 µl/well of blocking buffer (5% dry nonfat milk in 1x PBS, 0.05% Tween 20) and washed several times with washing buffer (1x PBS, 0.05% Tween 20). Phage clones (100 µl/well containing around 10^8^ plaque forming units in blocking buffer) were added to the plate and incubated for one hour at 37°C with gentle agitation. The serum pool was then serially diluted in blocking buffer and added to the wells (100 µl/well). Plates were incubated at 37°C for 1 h, washed and alkaline phosphatase-conjugated antibodies (Sigma-Aldrich, A9544) were added to each well. Para-nitrophenyl phosphate (Substrate 104; Sigma-Aldrich) was used to reveal enzymatic activity and absorbance at 405 nm was detected using an automated ELISA reader. Assays were performed in duplicate, and the mean values were calculated. Results were expressed as antibody titers, defined as the reciprocal of the highest serum dilution producing absorbance values higher than the mean +2 SD of those of wild type phage suspensions [41].

### Sequencing of isolated clones

Plaque picking and sequencing of isolated clones was performed as previously described [30], [40], [41]. Briefly, phage plaques from the output of the second selection round were randomly picked and, after amplification in bacteria, phage suspensions were partially purified by polyethylene-glycol/NaCl precipitation, as described [41], [42]. Purified suspensions were added to a PCR mix containing the above-listed K47 and K48 primers to amplify the insert-containing region and PCR products were analyzed by Sanger sequencing using capillary electrophoresis, as described (20, 21).

### Ethics statements

The studies have been conducted after approval of the review board of the Department of Experimental Pathology and Microbiology of the University of Messina (no. 12012012). Serum samples were obtained in an anonymized and de-identified form from Novartis Vaccines and Diagnostics.
